# Genetic Interactions Implicating Postreplicative Repair in Okazaki Fragment Processing

**DOI:** 10.1371/journal.pgen.1005659

**Published:** 2015-11-06

**Authors:** Jordan R. Becker, Carles Pons, Hai Dang Nguyen, Michael Costanzo, Charles Boone, Chad L. Myers, Anja-Katrin Bielinsky

**Affiliations:** 1 Department of Biochemistry, Molecular Biology, and Biophysics, University of Minnesota, Minneapolis, Minnesota, United States of America; 2 Department of Computer Science and Engineering, University of Minnesota, Minneapolis, Minnesota, United States of America; 3 Department of Molecular Genetics, University of Toronto, Toronto, Ontario, Canada; Duke University, UNITED STATES

## Abstract

Ubiquitination of the replication clamp proliferating cell nuclear antigen (PCNA) at the conserved residue lysine (K)164 triggers postreplicative repair (PRR) to fill single-stranded gaps that result from stalled DNA polymerases. However, it has remained elusive as to whether cells engage PRR in response to replication defects that do not directly impair DNA synthesis. To experimentally address this question, we performed synthetic genetic array (SGA) analysis with a ubiquitination-deficient K164 to arginine (K164R) mutant of PCNA against a library of *S*. *cerevisiae* temperature-sensitive alleles. The SGA signature of the K164R allele showed a striking correlation with profiles of mutants deficient in various aspects of lagging strand replication, including *rad27Δ* and *elg1Δ*. Rad27 is the primary flap endonuclease that processes 5’ flaps generated during lagging strand replication, whereas Elg1 has been implicated in unloading PCNA from chromatin. We observed chronic ubiquitination of PCNA at K164 in both *rad27Δ* and *elg1Δ* mutants. Notably, only *rad27Δ* cells exhibited a decline in cell viability upon elimination of PRR pathways, whereas *elg1Δ* mutants were not affected. We further provide evidence that K164 ubiquitination suppresses replication stress resulting from defective flap processing during Okazaki fragment maturation. Accordingly, ablation of PCNA ubiquitination increased S phase checkpoint activation, indicated by hyperphosphorylation of the Rad53 kinase. Furthermore, we demonstrate that alternative flap processing by overexpression of catalytically active exonuclease 1 eliminates PCNA ubiquitination. This suggests a model in which unprocessed flaps may directly participate in PRR signaling. Our findings demonstrate that PCNA ubiquitination at K164 in response to replication stress is not limited to DNA synthesis defects but extends to DNA processing during lagging strand replication.

## Author Summary

Genome duplication *via* the process of DNA replication is a prerequisite for cell division and underlies the propagation of all living organisms. This fundamentally important mechanism has been highly conserved throughout eukaryotic evolution, allowing us to use the relatively simple and genetically tractable *Saccharomyces cerevisiae* as a model to better understand DNA replication in human cells. Furthermore, there is strong evidence to suggest that defects in DNA replication are prominent contributors to mutation and genome instability, a hallmark of cancer. Not surprisingly, evolution has selected for mechanisms to mitigate the effects of defective replication and avoid the most harmful outcomes. Postreplicative repair (PRR) pathways are two such mechanisms with well described functions in promoting the completion of replication under adverse conditions. In this study, we utilized a non-biased genome wide genetic screen to systematically identify conditions under which PRR is required. Our findings indicate that in addition to previously described roles in rescuing DNA synthesis defects, PRR is also required in response to aberrant DNA processing. Specifically, we report a requirement for PRR in cells lacking *RAD27*, the yeast homolog of the tumor suppressor FEN1. These findings expand the known functions of PRR and reveal their importance in promoting the viability of cells lacking a known tumor suppressor.

## Introduction

The accurate copying of a cellular genome and subsequent transmission of genetic material to two daughter cells occurs on a microscopic scale, but is nonetheless a prodigious task. Considering the difficulty of accomplishing this fundamental process for living cells, it is hardly surprising that evolution has selected for a complex and multi-layered system of checkpoints and redundancies that promote its completion under sub-optimal conditions [1,2]. Many of these processes are regulated by post-translational modification of proteins that act as molecular switches to regulate downstream responses.

The replication clamp, proliferating cell nuclear antigen (PCNA), is one such target for a variety of post-translational modifications that trigger an array of downstream effects. Known modifications include the covalent attachment of ubiquitin and the small ubiquitin-like modifier (SUMO) to specific lysine (K) residues [3]. SUMO modification of chromatin-bound PCNA, or sumoylation, occurs during an unperturbed S phase at K164 and–to a lesser extent–at K127 [3]. Sumoylation acts primarily to recruit the helicase/anti-recombinase suppressor of rad six 2 (Srs2), which prevents illegitimate recombination at replication forks [4–6].

Ubiquitination of PCNA occurs predominately at K164, however, alternative attachment sites have been mapped in yeast and human cells [7–11]. In contrast to sumoylation, ubiquitination is induced by replication stress [3]. PCNA-K164 ubiquitination was initially identified as a response to template strand lesions, which stall the highly selective processive polymerases (Pol-) δ and Pol-ε [3]. Polymerase stalling leads to the accumulation of single-stranded (ss) DNA, which quickly becomes coated with replication protein A (RPA) [12]. This allows for the recruitment of the E2-E3 ubiquitin ligase complex radiation sensitive-6 and -18 (Rad6-Rad18) to mono-ubiquitinate PCNA-K164 [13]. Mono-ubiquitin can be subsequently extended to K63-linked poly-ubiquitin chains by methyl methanesulfonate sensitive 2-ubiquitin conjugating 13-radiation sensitive 5 (Mms2-Ubc13-Rad5) [3]. The length of the ubiquitin chain plays a crucial role in determining which of two PRR pathways is activated. Mono-ubiquitin facilitates an error-prone pathway for lesion bypass dependent on translesion polymerase activity [3,14–16]. Recent data indicates that replication past lesions by the mutagenic translesion polymerase Pol-ζ may continue for up to 1 kilobase beyond the lesion [17]. Alternatively, poly-ubiquitin chains enable a template switching pathway in which the nascent DNA of the sister chromatid acts as a template, allowing for lesion bypass and filling of ssDNA gaps [18]. This process is considered to be “error-free”, because it does not rely on the intrinsically mutagenic translesion polymerases of the error-prone pathway [19]. The precise mechanism of this branch is not yet well understood.

In addition to the originally described function of PRR in DNA damage tolerance and lesion bypass, recent work has suggested that mutants with impaired replisome function also activate these pathways for replication of undamaged template strands [20–23]. We have previously demonstrated that PRR promotes the viability of *mcm10* mutants in the absence of DNA damage [23]. To systematically explore the role of PCNA-K164 in response to intrinsic cellular dysfunction, we performed SGA analysis of a PCNA-K164 to arginine (PCNA-K164R) mutant. Interestingly, we found that the genetic interaction profile of the PCNA-K164R mutant closely resembled that of many alleles of lagging strand replication factors, including those involved in Okazaki fragment processing. This observation was particularly intriguing, as PRR has not been implicated in tolerating Okazaki fragment processing defects. As a result, we further investigated the activity of PCNA-K164-dependent pathways in mutants disrupting normal lagging strand replication. Specifically, we focused on the role of PCNA-K164 in cells deficient for the flap endonuclease radiation sensitive 27 (Rad27) or enhanced level of genomic instability 1 (Elg1), a homolog of replication factor C (RFC) subunit Rfc1 [24].

Rad27 processes 5’ flaps generated during lagging strand replication when DNA synthesis by Pol-δ collides with the 5’ end of the RNA-DNA primer of the previous Okazaki fragment, displacing it into a small <10-nucleotide (nt) flap [25,26]. If the 5’ flap escapes processing by Rad27 and grows long enough to bind replication protein A (RPA), it is then cleaved by Dna2 [26–28]. RPA binding of the flap serves both to inhibit Rad27, and to recruit Dna2 [27]. Dna2 cleaves the long ~30-nt flap to a short flap (5–10 nt), which can then be further processed by Dna2 or Rad27 into a ligatable nick [25,27–29]. Although processing of long flaps must be relatively efficient under normal conditions, *rad27Δ* mutants exhibit a temperature dependent slow-growth phenotype [30]. This is best explained by an increased rate in DNA replication and concomitant increase in the formation of long flaps [30,31]. At the restrictive temperature of 37°C, *rad27Δ* mutants are unable to meet flap processing demands, resulting in lethality, whereas at the semi-permissive temperature of 35°C growth is merely impaired [30,32]. In the absence of complete flap removal–even at lower temperatures–Pol δ-exonuclease (exo) activity can resect the nascent 3’ end allowing the small 5’ flap to re-anneal and form a ligatable nick [26,33]. After ligation of the nick by DNA ligase I (Cdc9), PCNA is unloaded from chromatin by the Elg1:Rfc2-5 complex [34–36]. In the present study, we report that PCNA is ubiquitinated in *rad27Δ* and *elg1Δ* mutants. Whereas ablation of PRR is inconsequential in the *elg1Δ* strain, both translesion synthesis (TLS) and template switching promote *rad27Δ* viability, possibly by enabling alternative flap processing. Furthermore, the long RPA-coated flaps generated in the absence of Rad27 play an active role in promoting the ubiquitination of PCNA at K164 and initiating PRR.

## Results

### PCNA-K164R mutants resemble lagging strand replication mutants

**Fig 1:**
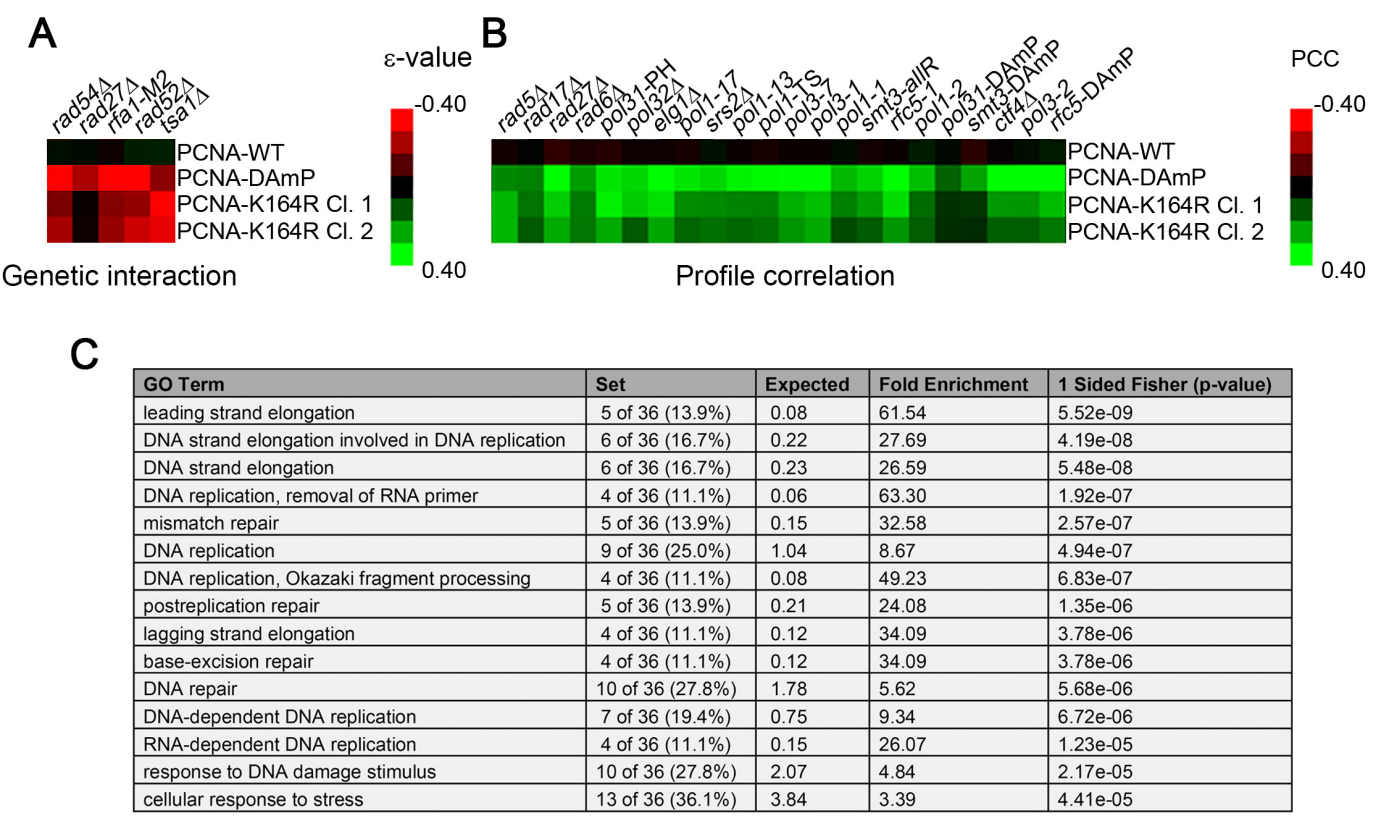
The PCNA-K164R SGA profile exhibits a limited set of direct genetic interactions but correlates strongly with other replication mutants. (A) Heat map of selected significant negative genetic interactions identified by SGA against the TS array for 2 independently isolated PCNA-K164R mutants. PCNA-WT and PCNA-DAmP are shown for comparison. The scale indicates epsilon- (ε-) values for the reported genetic interactions with negative interactions in red and positive interactions in green. (B) Heat map denoting the strength of correlation (measured by PCC) between PCNA-WT, PCNA-DAmP, PCNA-K164R clone 1 (Cl. 1) and PCNA-K164R clone 2 (Cl. 2) signatures against the TS array with the SGA signatures of the indicated strains. The scale denotes the strength of correlation between the indicated profiles with green being positive correlation and red being negative (C) Top 15 GO terms (http://www.ebi.ac.uk/QuickGO/) for alleles with PCC > 0.15 with the PCNA-K164R Cl. 1 profile against the FG array. This list is derived from a representative allele randomization (randomization 1 in S5 Table). Nine other randomizations were performed with similar results (S5 Table).

To examine the global role of PCNA-K164 in the absence of exogenous DNA replication stressors, we performed SGA analysis of two independently isolated PCNA-K164R mutant clones (identified as PCNA-K164R clone 1 and PCNA-K164R clone 2, respectively) against a library of temperature-sensitive (TS) alleles and a full genome (FG) array as previously described (S1–S4 Tables) [37,38]. Parallel analyses were performed against the TS array using a decreased abundance by mRNA perturbation (DAmP) allele of PCNA or a wild type (WT) allele as the query strain [39,40]. Since ubiquitination or sumoylation of K164 facilitates only a subset of PCNA functions, we anticipated that interactions identified in the PCNA-K164R SGA screens should represent a small part of those identified in the PCNA-DAmP analysis. Indeed the vast majority of hits identified in the K164R mutant screen with the TS array were also identified with the PCNA-DAmP allele (for PCNA-K164R clone 1: 18/26 hits overlapped with PCNA-DAmP with p-value < 10^−11^, and for PCNA-K164R clone 2: 11/14 hits overlapped with p-value < 10^−8^ as determined by Fisher’s test) (S1 and S2 Tables and Fig 1A). Furthermore, the negative genetic interactions were largely consistent with previous reports, including a requirement for PRR when thiol-specific antioxidant 1 (*TSA1*) is deficient [41]. Mutants of *TSA1* have reduced ability to neutralize reactive oxygen species, leading to increased DNA damage and synthetic sickness with PRR mutation [41]. We also observed a modest requirement for homologous recombination (HR) components in K164R mutants (Fig 1A) [42]. This gave us confidence that genes identified in the PCNA-K164R screens represented *bona fide* genetic interactions.

**Fig 2:**
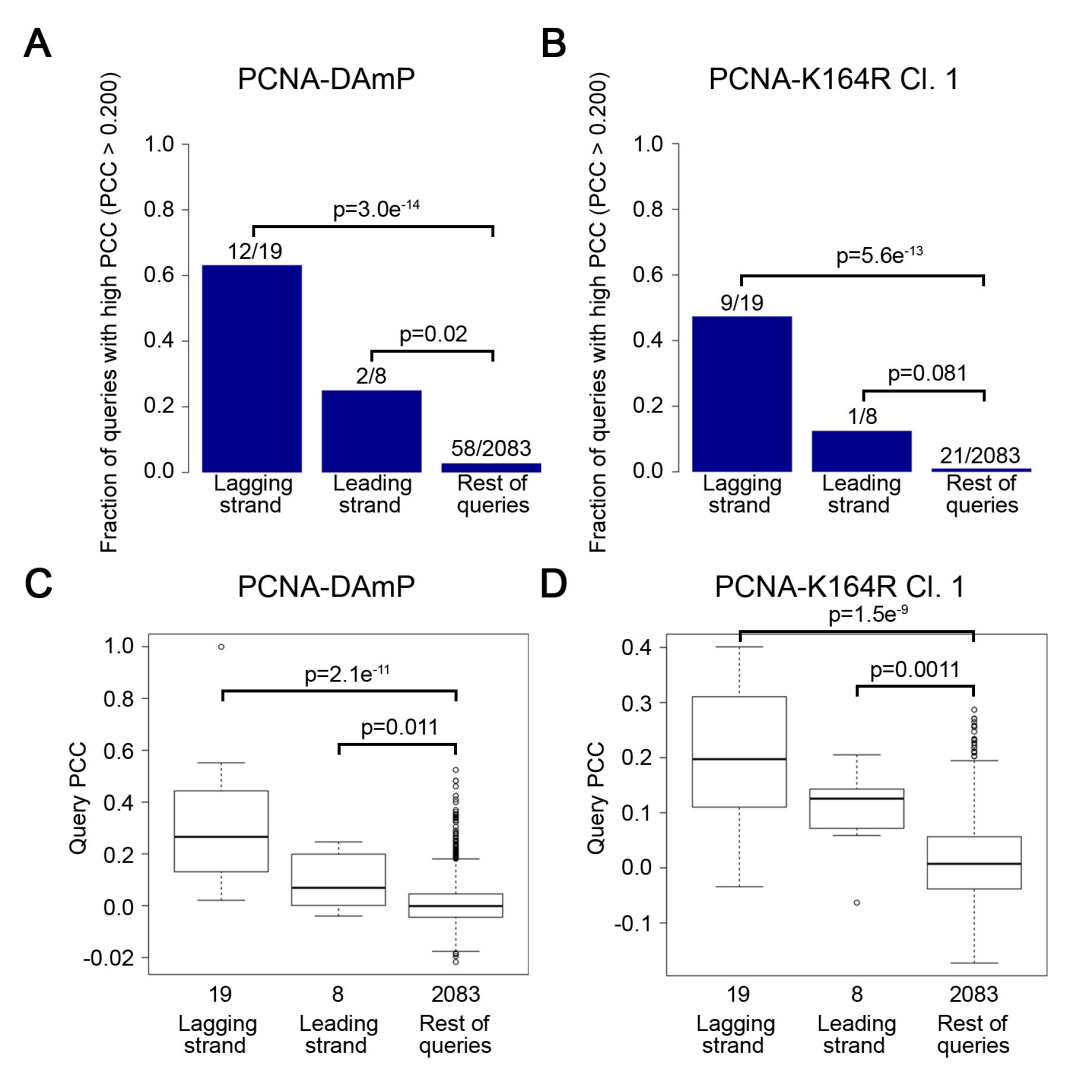
The PCNA-K164R SGA profile strongly resembles lagging strand replication mutant profiles. (A and B) The fraction of leading and lagging strand mutants with a similar profile (PCC > 0.2) to that of the PCNA-DAmP and PCNA-K164R alleles, respectively. PCNA-K164R Cl.1 was used for this analysis. All mutants were queried against the TS array. Significance was determined using Fisher’s exact test. (C and D) The distribution of profile similarities (calculated using PCC) between PCNA-DAmP or PCNA-K164R, respectively, and leading or lagging strand replication terms. All mutants were queried against the TS array for this analysis. Horizontal lines within the boxes indicate the median PCC. Error bars encompass the middle quartiles of the PCC value distribution. Outliers are represented with circular dots. Significance was determined by the Wilcoxon rank sum test [43].

The most informative results were revealed when examining the similarity of the PCNA-K164R SGA profiles with the interaction signatures of other mutants. Strong Pearson correlation coefficient (PCC) values between PCNA-K164R clones and *rad5Δ* and *rad6Δ* mutants were consistent with the known functions of K164 in facilitating PRR (Fig 1B and Table 1). Strikingly, PCNA-K164R also exhibited strong correlation with many mutant alleles of genes involved in lagging strand replication, suggesting that those mutants have replication defects similar to those in the PCNA-K164R mutants (Fig 1B and Table 1). To validate this observation in an unbiased manner, we performed a Gene Ontology (GO) enrichment analysis (http://www.ebi.ac.uk/QuickGO/). Alleles with SGA profiles similar to that of PCNA-K164R against the FG array (PCC > 0.15) were significantly associated with leading and lagging strand replication GO terms (Fig 1C). Interestingly, the list of enriched GO terms included “Okazaki fragment processing”, which has not been associated with PCNA-K164-dependent pathways (Fig 1C). GO enrichment of SGA profiles similar to PCNA-K164R against the TS array (PCC > 0.2) also showed nearly 25-fold enrichment with the “Okazaki fragment processing” term (S6 Table). Because this initial analysis relied on existing GO annotations, we manually compiled an informed list of genes associated with leading and lagging strand replication for further analysis (S7 Table). We found that PCNA-DAmP and PCNA-K164R profiles against the TS array were highly similar (PCC > 0.2) to profiles of genes implicated in leading and lagging strand replication (Fig 2A–2D). We confirmed these results when we compared the profile of a second PCNA-K164R query strain (Table 1 and S1 Fig). The PCNA-WT profile did not show any significant similarities (Table 1 and S1 Fig).

**Table 1:** Lagging strand replication mutants that correlate strongly with PCNA-DAmP and PCNA-K164R.

	PCNA-DAmP	PCNA-WT	PCNA-K164R Cl. 1	PCNA-K164R Cl. 2
***elg1Δ***	0.551	-0.023	0.356	0.262
***pol3-1***	0.511	-0.018	0.300	0.267
***rfc5-*DAmP**	0.494	0.041	0.296	0.190
***rad27Δ***	0.416	-0.077	0.325	0.268
***pol31-PH***	0.362	-0.058	0.375	0.269
***pol1-2***	0.300	0.045	0.170	0.147
***rad5Δ***	0.215	-0.029	0.287	0.288

PCC values for mutant alleles of lagging strand replication genes with TS array signatures that correlated strongly with PCNA-DAmP, PCNA-K164R Cl. 1, and PCNA-K164R Cl.2.

Altogether, these findings suggested that PCNA-K164 may have an active role in lagging strand replication, even in the absence of exogenous DNA damage. Strong correlations with PCNA-K164R included *pol1* mutants, which we previously described to activate PRR pathways, and *pol3* mutants (deficient in Pol-δ) which have also been described to elicit PCNA ubiquitination and TLS (Table 1) [20,23]. Additional strong correlations were observed for *rfc5*, *pol31*, *rad27*, and *elg1* mutants (Fig 1B and Table 1). These genes have been implicated in different steps of Okazaki fragment synthesis and processing, suggesting that PCNA-K164 is required at multiple junctions when lagging strand replication is impaired. This was surprising, as K164 ubiquitination of PCNA is dependent on the formation of ssDNA and is typically associated with DNA synthesis defects only [13]. The source of ssDNA–particularly in Okazaki fragment processing mutants, such as *rad27Δ* –was thus not immediately obvious. Nonetheless, these results led us to hypothesize that PCNA-K164-dependent pathways may be required to tolerate defects in lagging strand maturation. Because the function of K164 in PRR is dependent on its modification by ubiquitin, we hypothesized that lagging strand defects would result in chronic PCNA ubiquitination and activation of PRR pathways. To experimentally address this question, we assayed PCNA ubiquitination in *rad27Δ* and *elg1Δ* mutants, both of which had interaction profiles that correlated strongly with the PCNA-K164R alleles (Fig 1B and Table 1).

### *rad27Δ* and *elg1Δ* mutants constitutively ubiquitinate PCNA at K164

**Fig 3:**
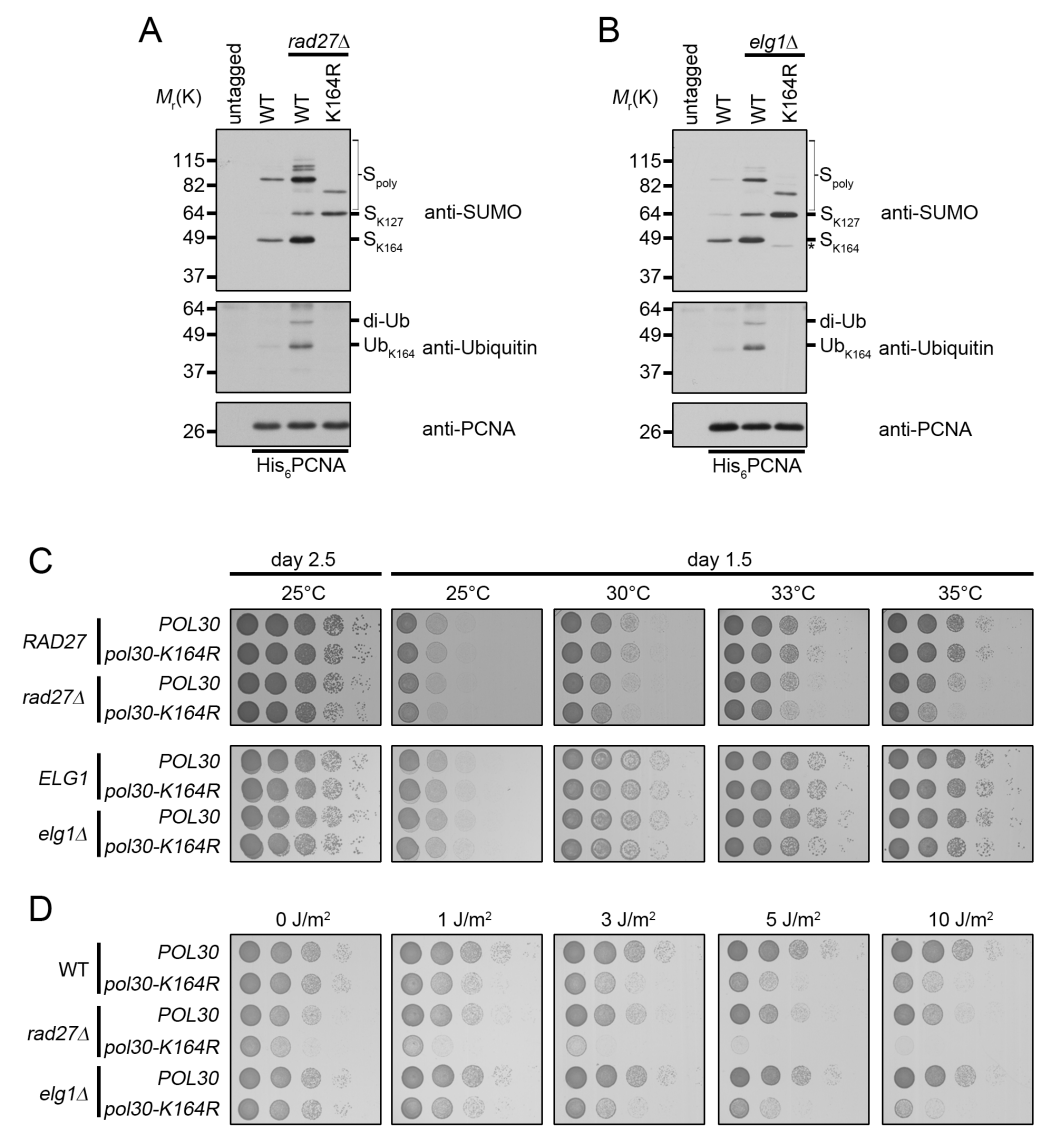
*rad27Δ* and *elg1Δ* mutants ubiquitinate PCNA at K164. (A) Asynchronous cultures were grown to OD_600_ = 0.600 at 25°C and then shifted to 37°C for 3 h before harvesting. His_6_-PCNA was purified under denaturing conditions and analyzed by western blot using specific antibodies against PCNA, ubiquitin, and SUMO as indicated. (B) Asynchronous cultures were grown to OD_600_ = 1.0 at 25°C before harvesting. PCNA was purified as in (A). The asterisk (*) indicates a minor PCNA-SUMO species observed in *elg1Δ pol30-K164R* mutants. (C) 10-fold serial dilutions of the indicated strains were incubated 1.5 or 2.5 days on YPD plates at varying temperatures. (D) 10-fold serial dilutions of the indicated strains were spotted on YPD plates and subsequently treated with UV. Plates were imaged 1.5 days after spotting.

*rad27Δ* is a temperature-sensitive allele, and for all subsequent experiments we shifted these mutants to 37°C for 3 h prior to analysis. To determine whether PCNA is ubiquitinated at K164 in *rad27Δ* and *elg1Δ* mutants, we purified histidine tagged PCNA (His_6_-PCNA) on Ni-NTA agarose and analyzed the eluates with PCNA-, ubiquitin- and SUMO-specific antibodies by western blot (Fig 3A and 3B). PCNA was indeed ubiquitinated in both mutants and ubiquitin attachment was completely ablated when PCNA carried a K164R substitution (Fig 3A and 3B), indicating that alternative attachment sites were not targeted. PCNA-K164R mutants also exhibited loss of K164-dependent sumoylation, consistent with previous reports [3,44]. Interestingly, when we introduced the PCNA-K164R mutation in *elg1Δ* cells, we reproducibly observed a minor PCNA-SUMO species of a slightly lower molecular weight than K164-SUMO (marked by an asterisk), consistent with an earlier study that revealed an alternative sumoylation site (Fig 3B) [44]. As shown previously, K127-SUMO migrated markedly slower than K164-SUMO. Moreover, levels of K127-SUMO were elevated in PCNA-K164R mutants [3]. Both *rad27Δ* and *elg1Δ* exhibited increased PCNA sumoylation at K127 and K164 compared to wild type (Fig 3A and 3B).

Next we asked whether PCNA-K164 modifications were important for viability of these two strains. Spotting analysis revealed that *rad27Δ* mutants had a significant reduction in growth at the semi-permissive temperature of 35°C when combined with the PCNA-K164R (*pol30-K164R*) mutation, whereas *elg1Δ* cells exhibited no such sensitivity at any temperature tested (Fig 3C), nor when they were exposed to UV light (Fig 3D). In contrast, *rad27Δ pol30-K164R* double mutants were acutely sensitive even to low doses of UV, showing a severe reduction in growth after exposure to 1J/m^2^ (Fig 3D). Together, these results suggested that K164-dependent pathways are important for the growth of *rad27Δ*, but not *elg1Δ* cells.

### TLS and template switching play redundant roles in *rad27Δ* mutants

**Fig 4:**
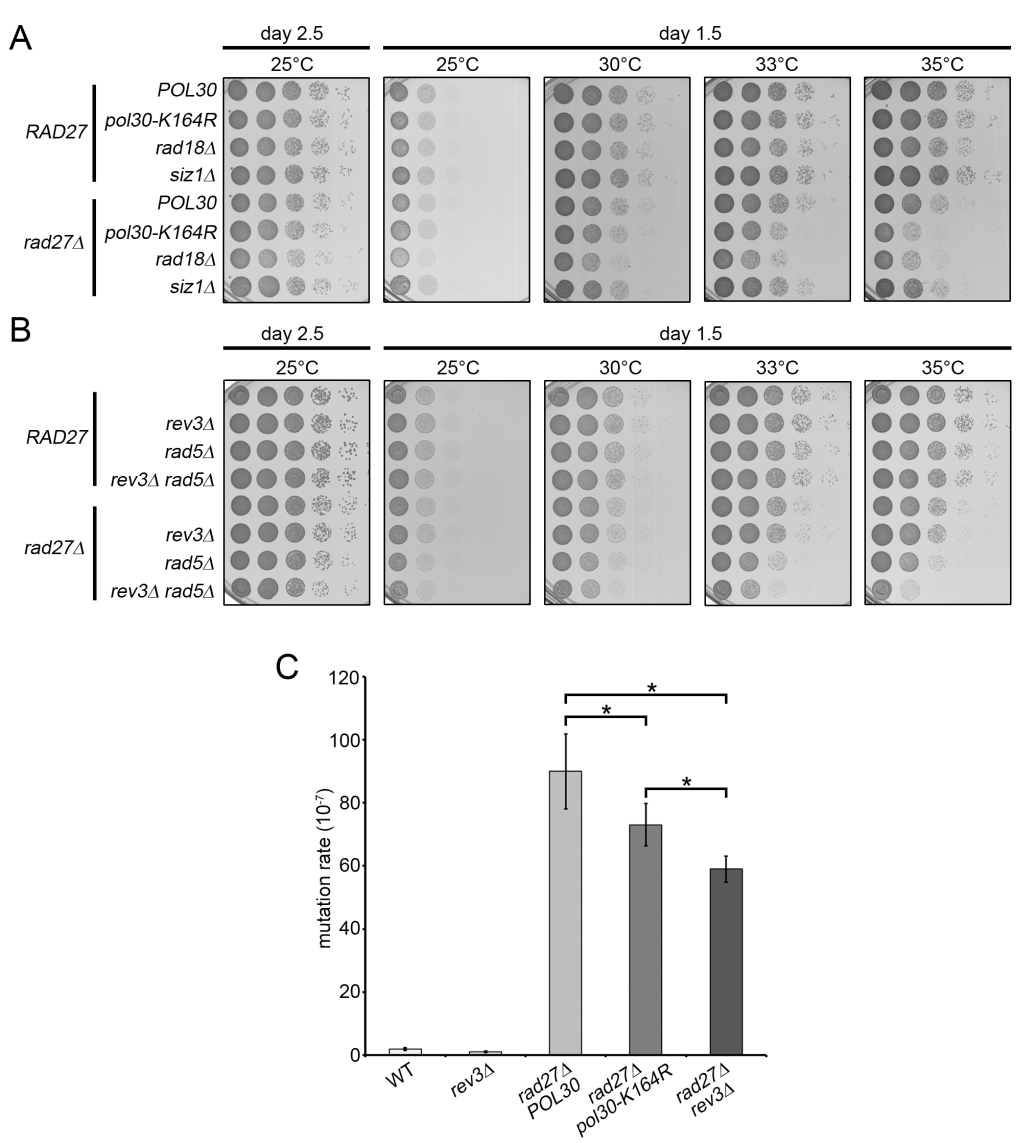
TLS and template switching are redundant in promoting *rad27Δ* viability. (A and B) 10-fold serial dilutions of the indicated strains were incubated 1.5 or 2.5 days on YPD plates at varying temperatures. (C) Bars indicate the rate of mutation at the *CAN1* locus for the indicated strains. Each measurement represents the median of at least 14 independent determinations. Significance was determined using the Mann-Whitney U test.

Because ubiquitination of PCNA at K164 is necessary for both TLS and template switching, we sought to determine which of these pathways are active in *rad27Δ* cells. Spotting analysis revealed that *pol30-K164R* and *rad18Δ* mutations each significantly reduced the viability of *rad27Δ* cells at 35°C (Fig 4A). The *rad27Δ rad18Δ* double mutant reproducibly appeared to have a slightly more severe growth defect than the *rad27Δ pol30-K164R* strain (Fig 4A). We attribute this finding to the known fact that PCNA-K164 sumoylation suppresses HR, and therefore substitution of K164 upregulates HR [4,5]. To address whether sumoylation of PCNA-K164 affected the viability of *rad27Δ* mutants, we deleted *SIZ1*, which encodes the SUMO E3 ligase that catalyzes this reaction. Consistent with published reports, *rad27Δ siz1Δ* double mutants did not exhibit any increased temperature sensitivity [42,45]. These results strongly suggest that the PCNA-K164 dependent phenotype is solely due to the loss of ubiquitination.

To estimate the relative contribution of TLS and template switching to *rad27Δ* viability, we generated *rad27Δ* strains with *rev3Δ* or *rad5Δ* mutations rendering them deficient in TLS and template switching, respectively. In addition, we analyzed a *rad27Δ rev3Δ rad5Δ* triple mutant, defective in both branches. We found that *rad27Δ rev3Δ* double mutants did not display any significant growth defects, whereas the *rad27Δ rad5Δ* cells exhibited a mild but noticeable growth delay, suggesting that the template switching pathway is the more prominent of the two (Fig 4B). However, loss of both pathways in the *rad27Δ rev3Δ rad5Δ* triple mutant resulted in a synergistic effect, resembling that of the *rad27Δ rad18Δ* double mutant (Fig 4A and 4B). These results argue that the two branches of PRR are both active in *rad27Δ* cells and have partially redundant roles in promoting viability.

The finding that *REV3* affected the survival of *rad27Δ* mutants in the absence of *RAD5* encouraged us to further examine the activity of the TLS branch of PRR. To accomplish this, we took advantage of the fact that TLS employs intrinsically mutagenic polymerases, which have a higher rate of nucleotide misincorporation combined with a lack of proofreading activity [46]. We predicted that TLS induced mutations would be dependent on K164. Mutation of K164 to arginine disables DNA synthesis by pol-ζ and its binding partner Rev1, which are responsible for the vast majority of TLS induced mutations [47]. Consistent with previous reports, fluctuation analysis revealed that *rad27Δ* mutants have a drastically increased rate of mutation (Fig 4C) [31,48]. Addition of the *pol30-K164R* allele led to a significant decrease in the mutation rate that accounted for 20–25% of total alterations, confirming that TLS was active in these cells (Fig 4C). Because the *pol30-K164R* mutation also removes the suppressive effect of PCNA-SUMO on HR, an increase in gross chromosomal rearrangements may mask the decrease in mutation rate due to the loss of TLS. Therefore, the K164-dependent reduction is likely an underestimation of the contribution by TLS [4–6]. In agreement with this notion, deletion of the pol-ζ catalytic subunit *REV3* results in a more severe reduction in the mutation rate than the *pol30-K164R* mutation (Fig 4C). Nonetheless, our observations are consistent with a recent report that had estimated point mutations in *rad27Δ* mutants to account for ~40% of all genomic aberrations [48]. Our results support the idea that the majority of these single nucleotide variations are a result of translesion polymerase activity.

### PCNA-K164 suppresses *rad27Δ* replication defects

**Fig 5:**
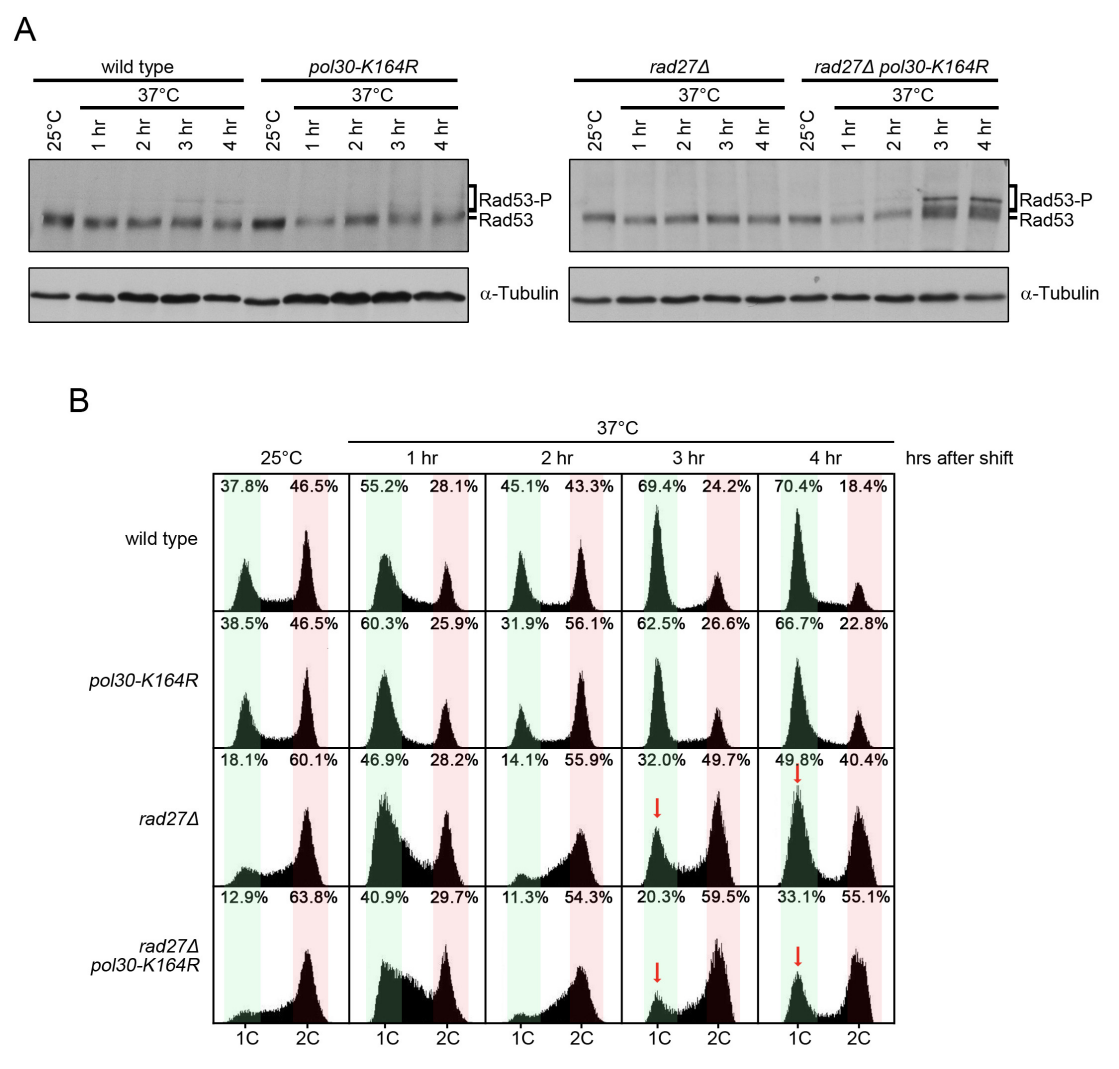
*rad27Δ pol30-K164R* double mutants exhibit increased checkpoint activation and cell cycle arrest. (A) The indicated strains were grown to OD_600_ = 0.600 at 25°C and then shifted to 37°C for 4 h. Samples were harvested immediately before the temperature shift and every hour for 4 h afterward. Protein was subsequently extracted by TCA precipitation. Extracts were fractionated by SDS-PAGE and analyzed by western blot with anti-Rad53 and anti-tubulin antibodies. Tubulin served as a loading control. (B) Aliquots of the same cultures from (A) were analyzed for DNA content by flow cytometry. Red arrows indicate the G1 peaks of *rad27Δ* and *rad27Δ pol30-K164R* after 3 and 4 h at 37°C for comparison. Percentages indicate the percent of all cells analyzed that fall within the highlighted area. Green regions indicate G1 phase peaks while red regions mark late S and G2/M phases.

To further explore how PCNA-K164 aids in cell survival, we analyzed activation of the Rad53 kinase, a downstream target of the mitotic entry checkpoint kinase 1 (Mec1), the homolog of human ATR (ataxia telangiectasia mutated- and Rad3-related) [49]. *rad27Δ pol30-K164R* double mutants showed increased phosphorylation of Rad53 relative to *rad27Δ* cells after they were shifted to the restrictive temperature of 37°C for 3 and 4 h. This was indicative of enhanced replication stress (Fig 5A). Consistently, the double mutants also displayed a robust late S/G2 phase arrest at those time points (Fig 5B). Since *rad27Δ* cells passed more proficiently through mitosis (indicated by the higher G1 peak marked with a red arrow at 3 and 4 h in Fig 5B), we concluded that PRR pathways likely facilitated the completion of S phase and ultimately allowed for entry into mitosis. Therefore, without PRR cells have a reduced ability to tolerate Rad27 deficiency. Altogether, our findings support a role for PRR pathways in suppressing replication defects when Rad27 is absent. In contrast, *elg1Δ pol30-K164R* double mutants did not display any Rad53 activation or observable alterations in cell cycle distribution relative to *elg1Δ* (S2 Fig).

### Overexpression of *EXO1* eliminated PCNA ubiquitination in *rad27Δ*

**Fig 6:**
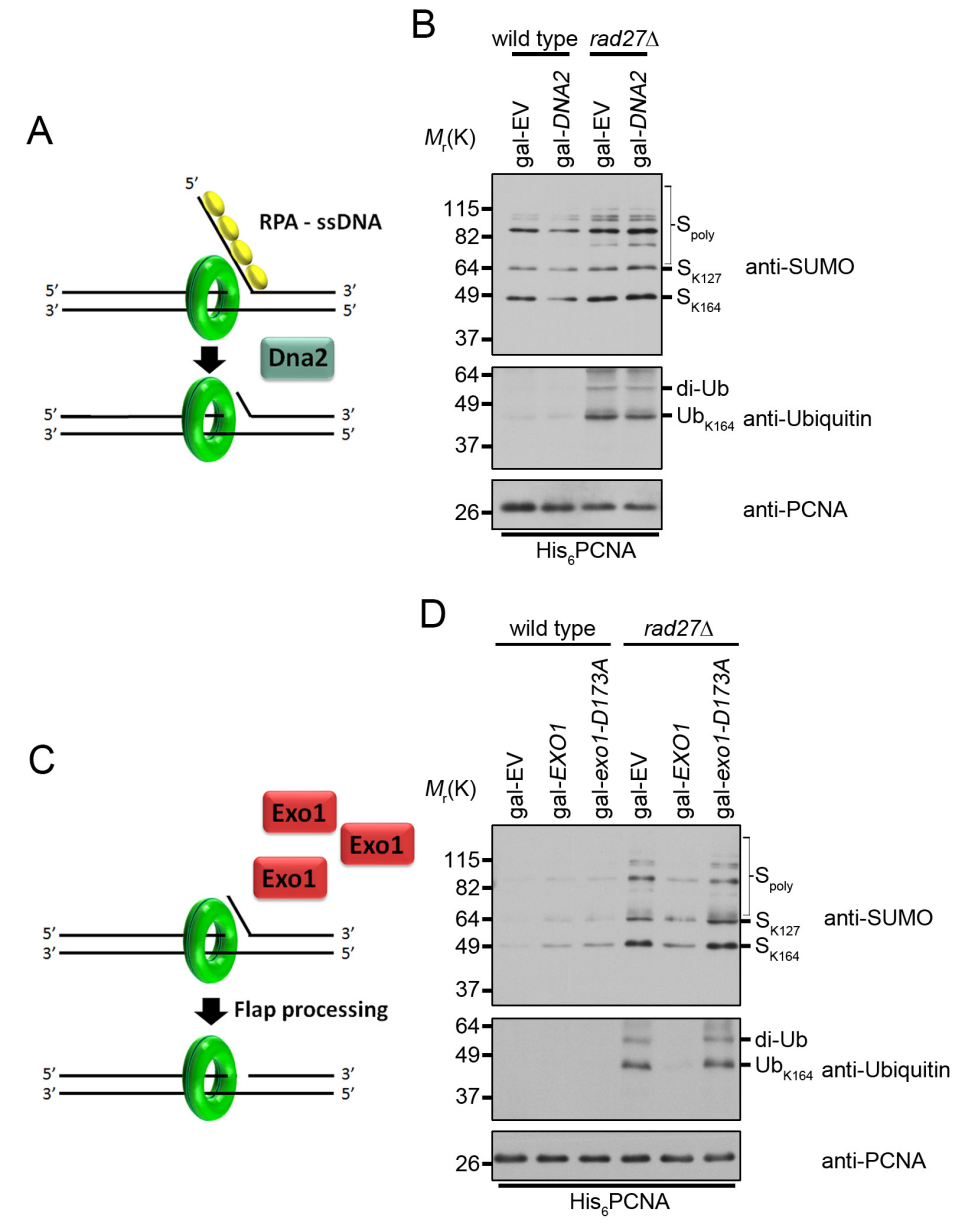
Overexpression of *EXO1* suppresses PCNA ubiquitination in *rad27Δ*. (A) RPA binding to long ssDNA flaps occurs *prior* to processing by Dna2. Therefore overexpression of *DNA2* is unlikely to interfere with RPA binding. (B) Wild type and *rad27Δ* cells carrying gal-EV or gal-*DNA2* plasmids were grown to OD_600_ = 0.600 at 25°C in raffinose containing medium lacking uracil. Galactose was then added to a final concentration of 2% and the cultures we shifted to 37°C for 3 h before harvesting. His_6_-PCNA was purified under denaturing conditions and analyzed by western blot with antibodies specific to PCNA, ubiquitin, and SUMO as indicated. (C) Cartoon depicting the effect of *EXO1* overexpression in the absence of flap endonuclease. If long RPA-coated flaps are the stimulus for PCNA ubiquitination in *rad27Δ*, we hypothesize that *EXO1* overexpression and processing of flaps before they are long enough to bind RPA will reduce ubiquitination. (D) Wild-type and *rad27Δ* cells carrying gal-EV, gal-*EXO1*, or gal-*exo1-D173A* plasmids were treated as in (B) in medium lacking uracil and purified PCNA was analyzed by western blot with antibodies specific to PCNA, ubiquitin and SUMO as indicated.

Previous work demonstrated that ubiquitination of PCNA at K164 by Rad6-Rad18 requires persistent regions of RPA-coated ssDNA [13]. These normally accumulate if the replicative polymerases are impeded [12]. However, in the context of Rad27 deficiency, the source of ssDNA was not readily apparent. Earlier studies established that in the absence of Rad27, Okazaki fragment flaps are processed through a “long flap” pathway by the combined activities of Dna2 and Pol3-exo [25,26,33,50,51]. In this pathway short flaps become longer through enhanced strand displacement until they are sufficiently large to be bound by RPA [52,53]. Binding by RPA serves to recruit Dna2 and stimulate its nuclease activity, reducing the flap to approximately 5 nt [27,28]. Although Dna2 has been shown to be competent to subsequently cleave the remaining short flap *in* vitro [28], Pol3-exo activity is clearly essential in *rad27Δ* mutants at all temperatures [33]. Pol3-exo is thought to resect the 3’ end of the preceding Okazaki fragment, allowing the remaining 5’ flap to re-anneal and form a ligatable nick [26,33]. It is conceivable that both Dna2 and Pol3-exo contribute to the resolution of short flaps in *rad27Δ* cells. The binding of RPA to long flap intermediates *prior* to processing by Dna2 led us to consider that long ssDNA flaps themselves could provide the stimulus for PCNA ubiquitination. We inferred that the close proximity of these RPA-bound structures to PCNA should allow for recruitment of the Rad6-Rad18 complex and subsequent PCNA ubiquitination. To test this hypothesis, we sought to modulate flap processing by overexpression of *DNA2* [29]. Because the current model for Dna2 processing of 5’ flaps proposes that RPA binding occurs prior to cleavage, we expected that recruitment of Rad6 and Rad18 may not be significantly reduced upon *DNA2* overexpression (Fig 6A) [27,54], unless it would directly compete with the E2-E3 complex. Notably, overexpression of *DNA2* did not reduce PCNA ubiquitination in *rad27Δ* (Fig 6B). We also considered the possibility that Pol3-exo activity during long flap processing could generate ssDNA regions sufficiently large to bind RPA (S3A Fig) However, overexpression of an exonuclease-dead allele of *POL3* (*pol3-01*) failed to reduce PCNA ubiquitination in *rad27Δ* (S3B Fig). Consistent with previous reports, *pol3-01* expression was lethal in combination with *rad27Δ* (S3C Fig) [55].

We next sought to modulate flap processing in a manner that reduced the formation of long RPA-bound flaps. Multiple studies have demonstrated that overexpression of exonuclease 1 (*EXO1*) rescues the DNA damage sensitivity of *rad27Δ* mutants [56–59]. Exo1 and Rad27 are both Rad2 family nucleases and crystal structures of their human homologs, FEN1 and EXO1, reveal highly conserved mechanisms of substrate binding and cleavage [60–62]. Thus, we hypothesized that Exo1, like Rad27, may cleave flaps *before* RPA can bind to them. If this were true, Exo1 overexpression should reduce PCNA ubiquitination in *rad27Δ* cells (Fig 6C). Indeed, overexpression of *EXO1* from a galactose inducible promoter eliminated PCNA ubiquitination in *rad27Δ* mutants (Fig 6D). Furthermore, this phenotype was dependent on the catalytic activity of *EXO1*, as overexpression of a nuclease-dead *exo1-D173A* allele had no impact on PCNA ubiquitination (Fig 6D) [59,63]. Furthermore, *EXO1* overexpression did not rescue the temperature sensitivity of *rad27Δ* (S4 Fig). In summary, our results suggest that the majority of PCNA ubiquitination in *rad27Δ* is dependent on RPA-coated ssDNA intermediates, which recruit the Rad6-Rad18 complex and are degraded by Exo1.

### PCNA ubiquitination caused by ssDNA gaps is not impacted by EXO1 overexpression

**Fig 7:**
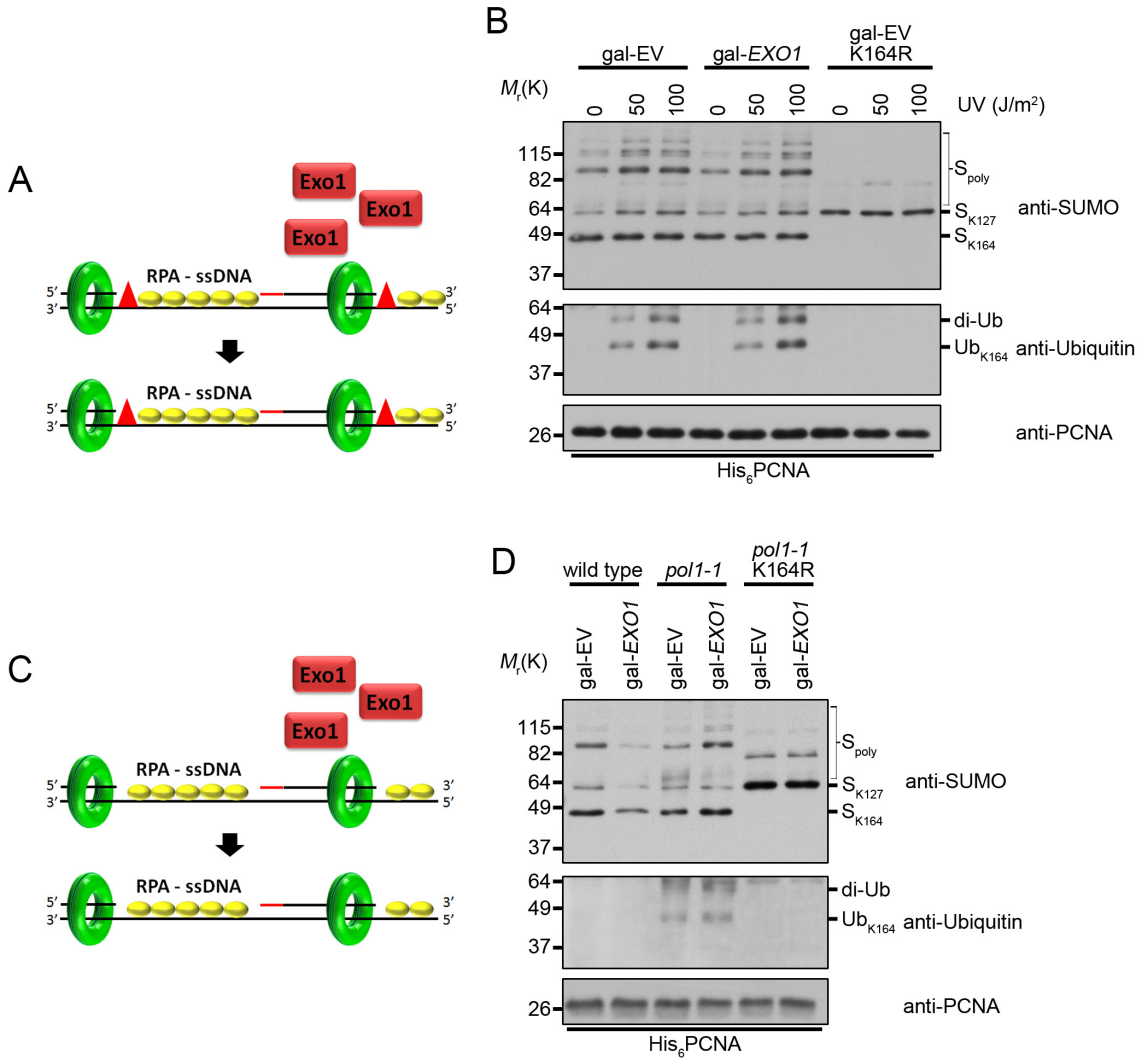
Overexpression of *EXO1* does not alter PCNA ubiquitination under conditions that cause ssDNA gap formation. (A) UV treatment leads to the formation of ssDNA gaps in replicating cells [12]. Overexpression of *EXO1* has no impact on ssDNA gap formation. The red triangles indicate UV-induced lesions. RPA-coated ssDNA is marked as RPA-ssDNA. (B) Cells carrying gal-EV or gal-*EXO1* plasmids were grown to OD_600_ = 0.600 at 25°C in raffinose containing medium lacking uracil. Galactose was then added to a final concentration of 2% and the cells were grown an additional 2 h at 25°C. Each culture was then split into 3 parts and treated with either 0, 50, or 100 J/m^2^ UV and left to recover for 40 min at 25°C before harvesting. His_6_-PCNA was purified under denaturing conditions and analyzed by western blot with antibodies specific to PCNA, ubiquitin, and SUMO as indicated. (C) Inefficient priming along the lagging strand template leads to the formation of RPA-coated ssDNA gaps (RPA-ssDNA) in *pol1* mutants [23,64,66]. Overexpression of *EXO1* has no impact on ssDNA gap formation. (D) Wild-type and *pol1-1* cells carrying gal-EV or gal-*EXO1* plasmids were grown to OD_600_ = 0.600 at 25°C in raffinose containing medium lacking uracil. Galactose was then added to a final concentration of 2% and the cultures we shifted to 35°C for 3 h before harvesting. His_6_-PCNA was purified under denaturing conditions and analyzed by western blot with antibodies specific to PCNA, ubiquitin, and SUMO as indicated.

To examine whether the effect of *EXO1* overexpression on PCNA ubiquitination in *rad27Δ* mutants could be due to indirect suppression of ssDNA gap formation, we exposed *EXO1* overexpressing cells to 50 J/m^2^ of UV light, which has been proven to cause ssDNA gaps (Fig 7A) [12]. Overexpression of *EXO1* had no impact on the level of PCNA ubiquitination under these conditions (Fig 7B). Moreover, we did not observe any differences in ubiquitination when cells were treated with 100 J/m^2^ of UV light, arguing that Exo1 did not act directly or indirectly to eliminate ssDNA regions (Fig 7B). In support of this conclusion, overexpression of *EXO1* had no impact on PCNA ubiquitination in cells harboring the temperature sensitive *pol1-1* allele (Fig 7C and 7D). This allele is thought to generate ssDNA regions as a result of reduced efficiency in the priming of Okazaki fragments (Fig 7C) [64,65], and causes ubiquitination of PCNA at K164 at the non-permissive temperature of 35°C [23]. Taken together, these findings indicate that Exo1 does not suppress the formation of ssDNA gaps.

## Discussion

The power of SGA analysis to identify networks of genetic interactors has greatly increased our knowledge of cellular pathway control and carries the considerable advantage of being an unbiased systems-level approach to complex biological questions [67]. However, such screens are useful not only for identifying direct genetic interactors, but also in revealing functional relationships between genes through comparison of their SGA signatures [37,38,68]. Using this type of comparative analysis we identified a pattern of correlation between the profiles of PCNA-K164R mutants and the profiles of several lagging strand replication and Okazaki fragment processing mutants, including *rad27Δ* (Fig 1B and Table 1). The high degree of similarity between the *rad27Δ* and PCNA-K164R profiles suggested to us that PCNA-K164-mediated pathways may counteract replication defects in *rad27Δ*. Proof of PCNA ubiquitination in *rad27Δ* cells and synthetic sickness in *rad27Δ pol30-K164R* and *rad27Δ rad18Δ*, but not *rad27Δ siz1Δ* double mutants further substantiated this notion and led us to focus on the role of K164 ubiquitination-dependent pathways in the absence of flap endonuclease (Figs 3A and 4A).

### Long ssDNA flaps are a platform for PRR activation

The primary replication defect in *rad27Δ* cells is caused by impaired processing of 5’ flaps generated during Okazaki fragment processing [31]. At elevated temperatures, DNA replication proceeds more rapidly, likely leading to an increase in the formation of long flap intermediates, which must bind RPA before they can be efficiently processed [27]. We speculated that these long RPA-coated flaps may serve as a platform to promote PCNA ubiquitination. To examine this hypothesis, we modulated flap length by overexpression of *EXO1*, a close relative of *RAD27* with a highly conserved mechanism of substrate binding and cleavage [60,61]. A number of prior studies have demonstrated that overexpression of *EXO1* suppresses the intrinsic mutagenicity of the *rad27Δ* allele [56,57]. In particular, *EXO1* overexpression suppresses the duplication of short direct repeats that have been hypothesized to result from longer flap structures generated in *rad27Δ*, which is consistent with Exo1 activity preventing long RPA-coated flap formation [56,57]. Thus, our finding that catalytically active Exo1 counteracts PCNA ubiquitination in *rad27Δ*, but has no effect on ubiquitination in *pol1-1* cells or after UV treatment, argues that the DNA structures mediating ubiquitination in flap endonuclease deficient cells are different from ssDNA gaps (Figs 6 and 7).

It has been speculated that long unprocessed flaps could participate in the replication stress response [10,52,69]. Campbell and colleagues found that constitutive Mec1 activation is responsible for *dna2Δ* lethality [69]. They hypothesized that Mec1 activation originated from long RPA-coated flaps that accumulate in these mutants [69]. Interestingly, *EXO1* overexpression partially rescues the temperature sensitivity of a viable *dna2-1* mutant, consistent with the notion that Exo1 acts upstream of long flap formation [70].

Another well-documented hallmark of Okazaki fragment processing mutants is profound instability of trinucleotide repeat (TNR) regions [71,72]. This raises the question as to whether a requirement for PCNA-K164 in *rad27Δ* mutants is specific to the replication of TNR regions. A previous study had linked error-free PRR to the suppression of TNR expansion in *rad27Δ* cells [73]. However, error-prone TLS did not appear to regulate TNR expansion at all [73,74]. Our finding that both TLS and error-free PRR are active in *rad27Δ* cells therefore leads us to conclude that the role of PCNA-K164 in these mutants is not restricted to genomic regions that encompass TNRs (Fig 4B).

### PCNA ubiquitination is a sensor of Okazaki fragment processing defects

Historically, PCNA ubiquitination and PRR were considered rescue pathways for template strand lesions that impair polymerase progression and require TLS or template switching for bypass [3]. Later work from the Shcherbakova group demonstrated that intrinsic replisome deficiencies in hypomorphic alleles of the replicative polymerases *POL2* and *POL3* also lead to PCNA ubiquitination and activation of TLS on undamaged DNA templates [20]. This important finding described ubiquitination of PCNA and activation of PRR in the absence of replication stressors that damage DNA. Nevertheless, the essential effect of DNA damaging agents and hypomorphic polymerases on replication is a disruption of DNA synthesis. Both therefore intuitively lead to ssDNA gaps, triggering PCNA ubiquitination and subsequent gap-filling by PRR.

In contrast, our study describes PCNA ubiquitination under conditions in which DNA synthesis is not impaired. Rad27 catalyzes Okazaki fragment flap cleavage, which does not occur until *after* Okazaki fragment synthesis, yet we see a requirement for PRR to support the viability of Rad27 deficient cells. This distinction suggests that PCNA-K164 is active in mediating DNA processing defects that are unlinked to problems in DNA synthesis. It is tempting to speculate that PRR pathways are promoting processing of Okazaki fragments by Dna2 or potentially an alternative mechanism. Error-free template switching could allow for synthesis past multiple Okazaki fragments using the sister chromatid as a template and reducing the overall requirement for flap endonuclease. A role for PRR in promoting flap processing and thereby reducing the half-life of long flaps is consistent with our observation of elevated Rad53 phosphorylation in *rad27Δ pol30-K164R* double mutants (Fig 5A). The mechanism by which PRR promotes flap processing or bypass is currently unclear.

### PRR does not sustain survival of *elg1Δ* mutants

Similar to *RAD27*, *ELG1* has been described as an important protector of genome stability [24,75–78]. Recent reports have identified Elg1 as a crucial component of an alternative RFC complex that unloads PCNA from double-stranded DNA [34,35]. Our SGA screen revealed that the genetic interaction profile of *elg1Δ* correlates strongly with the PCNA-K164R and PCNA-DAmP profiles, leading us to investigate ubiquitination of PCNA at K164 in this mutant (Figs 1B and 3B and Table 1). Unlike *rad27Δ*, *elg1Δ* cells did not exhibit synthetic sickness with the K164R mutation and displayed no acute requirement for PRR pathways to tolerate intrinsic replication stress or mild UV treatment (Fig 3C and 3D). We speculate that replication defects in *elg1Δ* are mimicking those present in PCNA-DAmP mutants in that both are limiting the amount of free PCNA in the nucleus that is available to load onto chromatin and engage in replication. In the case of the PCNA-DAmP allele this is simply the result of lower steady-state levels of PCNA protein, whereas in *elg1Δ*, PCNA is likely sequestered on DNA due to diminished unloading [34,35,79].

In summary, our results suggest that during the processing of Okazaki fragments *via* the long flap pathway, the flap itself is likely not an inert DNA processing intermediate, but may play an active role in signaling replication stress through PCNA. It is conceivable that under normal conditions a division of labor between long and short flap processing is required for efficient Okazaki fragment maturation [27,80–82]. In *rad27Δ* cells, the balance is pushed severely to the long flap pathway, leading to the accumulation of RPA bound ssDNA structures that can be eliminated by Exo1. This becomes particularly problematic at elevated temperatures when the kinetics of DNA replication are increased and flaps are produced at a higher rate. The mechanism by which PRR is suppressing replication stress under these conditions is not clear at this time, but we speculate that it is helping to circumvent flap processing.

These findings are salient in light of the relationship between the regulation of flap processing and cancer. Homozygous deletion of the *RAD27* homolog FEN1 in mice is lethal, but heterozygous deletion in combination with mutation of the adenomatous polyposis coli (Apc) gene results in increased numbers of adenocarcinomas, enhanced tumor progression and decreased survival [83]. Mutations which reduce FEN1 activity have also been demonstrated to vastly increase cancer incidence in mouse models [84]. This study provides molecular evidence for the pathways contributing to mutagenesis when flap endonuclease function is compromised and gives insight into how cells sustain viability under these conditions.

## Materials and Methods

### Strains and plasmids

All yeast strains used in this study are isogenic derivatives of wild type E133 cells, which were derived from CG379 [85], with the exception of *pol1-1* strains which are derived from SSL204 [23]. Strains with gene deletions were generated by PCR mediated gene disruption [86]. All clones were verified by PCR and sequencing of the modified locus. Strains carrying *pol30-K164R* mutations were generated by PCR mediated gene disruption as follows: continuous PCR fragments consisting of PCNA, its endogenous promoter and a *LEU2* marker were amplified from pCH1654 (a gift from L Prakash, UTMB) and integrated at the endogenous PCNA locus. Integration and clonal homogeneity were verified by PCR and sequencing. All strains generated in this study are listed in S8 Table.

His_6_-tagged PCNA strains were constructed using Yip128-P30-POL30wt (gift from HD Ulrich, IMB Mainz). Plasmid variants with lysine mutations were constructed using the QuikChange Lightning (Agilent Technologies) site-directed mutagenesis kit. Briefly, the plasmid was linearized at an AflII restriction site in the *LEU2* coding sequence and transformed. Clones were screened by PCNA western blot to ensure that His_6_-PCNA expression was equivalent to endogenous (untagged) expression levels. The endogenous copy of PCNA was then knocked out via PCR mediated gene disruption.

In experiments using galactose inducible gene expression, liquid cultures were grown to OD_600_ = 0.600 at 25°C in raffinose containing medium. Galactose was then added to a final concentration of 2% and the cultures were shifted to 37°C for 3 h before collecting. Overexpression of *POL3* and *pol3-01* was induced by adding galactose to cells carrying the plasmids pBL336 and pBL336-01, respectively (gifts from D. Gordenin, NIEHS, originally constructed in P.M.J. Burgers laboratory, Washington University in St. Louis) [26]. Expression of *DNA2* was induced with galactose in cells carrying pgal-*DNA2* (a gift from R. Wellinger, Université de Sherbrooke, Québec) [87]. *EXO1* overexpression was induced by adding galactose to cells carrying pcDNA50.1, a derivative of pRS316 that was referred to as gal-*EXO1* (a gift from K. Lewis, Texas State University) [58]. The *exo1-D173A* variant was generated using the QuikChange Lightning (Agilent Technologies) site-directed mutagenesis kit.

UV treatment (254nm) of liquid cultures was applied using a UV crosslinker (CL-1000, UVP). Cultures were transferred to a sterile tray and treated in the crosslinker before being returned to flasks and cultured an additional 40 min before harvesting.

### Synthetic Genetic Array (SGA) screen

A genome-wide screen for genetic interactions with four *POL30* alleles as query strains (PCNA-DAmP, PCNA-WT, PCNA-K164R Cl.1, and PCNA-K164R Cl.2) was conducted at 30°C as previously described [38]. Because the screens are performed at 30°C they did uncover a synthetic interaction between PCNA-K164R and *rad27Δ*. Briefly, the query strains, marked with a nourseothricin (*NatMX4*) resistance cassette and harboring the SGA haploid specific markers and reporter [38], were mated to an array of 786 temperature-sensitive and 175 viable deletion mutants (TS array: manuscript in preparation) (S1 and S2 Tables). Additionally, PCNA-K164R Cl.1 and Cl.2 were mated to an array of 3827 viable deletion mutants (FG array). Nourseothricin- and geneticin-resistant heterozygous diploid mutants were selected and sporulated with *MATa pol30* double mutants as described [38]. Results of this screen are also included in the supplementary material (S3 and S4 Tables). Different PCC cutoffs were applied to the FG and TS array data (0.15 and 0.2, respectively) in order to enrich for the top 2% of all profile correlations.

### His_6_-PCNA purification

Cultures were grown to OD_600_ = 0.600 at 25°C and then shifted to 37°C for 3 h before harvesting (with the exception of *elg1Δ* strains which remained at 25°C for 3 h after reaching OD_600_ = 0.600). Cell pellets were frozen at -80°C. Briefly, cells were lysed under denaturing conditions and protein extracts were prepared as previously described [88]. Extracts were incubated rotating overnight at room temperature with Ni-NTA conjugated agarose (Qiagen) to bind His_6_PCNA. After incubation, His_6_PCNA-bound beads were washed with buffers of decreasing pH to increase stringency with successive washes. His_6_-PCNA was eluted from the beads with an EDTA-containing buffer and eluates were fractionated by SDS-PAGE. Purified PCNA and modified forms were then analyzed by western blot using specific antibodies against PCNA, ubiquitin, and SUMO.

### Protein extraction and western blotting

Whole cell protein extraction was accomplished by TCA precipitation as previously described and fractionated by SDS-PAGE [89]. Western blots were probed using the following antibodies; anti-PCNA at 1:4000 dilution (S871, a gift from B. Stillman, CSHL), anti-SUMO at 1:3000 dilution (A gift from X. Zhao, MSKCC), anti-ubiquitin at 1:1000 dilution(P4D1, Covance), anti-Rad53 at 1:1000 dilution (A gift from JFX Diffley, LRI, UK), anti-phospho-S129 H2A at 1:1000 dilution (ab15083, Abcam), and anti-tubulin at 1:5000 dilution (MMS-407R, Covance).

### Mutation rate analysis

Mutation rates were estimated by measuring the forward rate of mutations at the *CAN1* locus that confer resistance to canavanine [90]. Determinations were made from the median of at least 14 independent cultures for each strain. Cultures were inoculated from single colonies in 5 ml of YPD medium and grown to saturation for 5 days at 30°C. Cells were then washed and diluted to appropriate concentrations before plating on medium lacking arginine and containing canavanine (60 mg/L). Dilutions were also plated on non-selective YPD to obtain a viable cell count. Mutation rates were calculated using Drake’s formula as previously described [91,92]. Significance was determined by the Mann Whitney U test as previously described [43].

### Cell viability analysis

Relative cell viability was measured using an assay referred to as the “spotting assay”. In this assay, 10 ml cultures were grown to saturation for 4 days at 25°C. Cells were then harvested, washed with water, quantified, and diluted to equal volumes containing 2x10^7^ cells. 10-fold serial dilutions were made from these 2x10^7^ cells in a 96-well plate and then “spotted” on rich medium using a multi-pronged spotting manifold. Replicates were generated for incubation at various temperatures. UV treatment (254nm) was applied where indicated after plating using a UV crosslinker (CL-1000, UVP). Plates were imaged after 1.5 days of growth except where indicated.

### Cell cycle analysis

Cell cycle progression was measured by flow cytometry as previously described [8]. Briefly, 1 ml of liquid culture was pelleted and fixed in ice-cold 70% ethanol overnight. DNA was stained with Sytox Green (Invitrogen) and cells were analyzed using a BD Accuri C6 flow cytometer (BD Biosciences). Peaks were quantified using the quantification feature of the BD Accuri C6 software.

## Supporting Information

S1 Fig The PCNA-K164R Cl. 2 SGA profile, but not that of PCNA-WT, strongly resembles the genetic interactions of lagging strand replication mutants. (A and B) The fraction of leading and lagging strand mutants with a similar profile (PCC > 0.2) to that of the PCNA-WT and PCNA-K164R alleles, respectively. PCNA-K164R Cl.2 was used for this analysis. All mutants were queried against the TS array. Significance was determined using Fisher’s exact test. (C and D) The distribution of profile similarities (calculated using PCC) between PCNA-WT or PCNA-K164R, respectively, and leading or lagging strand replication terms. PCNA-K164R Cl.2 was used for this analysis. All mutants were queried against the TS array. Horizontal lines within the boxes indicate the median PCC. Error bars encompass the middle quartiles of the PCC value distribution. Outliers are represented with circular dots. Significance was determined by the Wilcoxon rank sum test [43].
(TIF)

S2 Fig *elg1Δ pol30-K164R* double mutants do not exhibit increased replication defects. (A) The indicated strains were grown to OD_600_ = 0.600 at 25°C and then split in half, either remaining at 25°C or being shifted to 37°C. Both cultures were harvested after 3 h growth and protein was extracted by TCA precipitation. Extracts were fractionated by SDS-PAGE and analyzed by western blot with antibodies specific to Rad53 and phospho-H2A-S129. Tubulin served as a loading control. (B) Aliquots of the same cultures from (A) were analyzed for DNA content by flow cytometry.
(TIF)

S3 Fig Overexpression of *pol3-01* does not suppress PCNA ubiquitination in *rad27Δ*. (A) Long flaps generated in the absence of Rad27 are processed into short flaps before Pol3-exo activity resects the 3’ end of the nascent DNA strand allowing for the short flap to re-anneal and form a ligatable nick. If 3’ resection is extensive enough to form a ssDNA region sufficient to bind RPA we considered that this could serve as the stimulus for PCNA ubiquitination in *rad27Δ*. (B) Wild type and *rad27Δ* cells carrying gal-EV, gal-*POL3*, or gal-*pol3-01* plasmids were grown to OD_600_ = 0.600 at 25°C in raffinose containing medium lacking tryptophan. Galactose was then added to a final concentration of 2% and the cultures were shifted to 37°C for 3 h before harvesting. His_6_-PCNA was purified under denaturing conditions and analyzed by western blot with antibodies specific to PCNA, ubiquitin, and SUMO as indicated. **(C)** 10-fold serial dilutions of the indicated strains were incubated 3 days at 35°C on medium lacking tryptophan and containing either 2% glucose or 2% galactose.
(TIF)

S4 Fig *EXO1* overexpression does not rescue the temperature sensitivity of *rad27Δ* mutants. 10-fold serial dilutions of the indicated strains were incubated 3 days at 25°C or 35°C on medium lacking uracil and containing either 2% glucose or 2% galactose.
(TIF)

S1 Table Full results of SGA screens with PCNA-DAmP, PCNA-WT, PCNA-K164R Cl.1 and PCNA-K164R Cl.2 against the TS array. (XLSX)

S2 Table Negative genetic interactions with PCNA-DAmP, PCNA-WT, PCNA-K164R Cl.1 and PCNA-K164R Cl.2 against the TS array. (XLSX)

S3 Table Full results of SGA screens with PCNA-K164R Cl.1 and PCNA-K164R Cl.2 against the FG array. (XLSX)

S4 Table Negative genetic interactions with PCNA-K164R Cl.1 and PCNA-K164R Cl.2 against the FG array. (XLSX)

S5 Table Allele randomizations for GO analysis of FG array results for PCNA-K164R Cl.1. (XLSX)

S6 Table GO enrichments for PCNA-K164R Cl.1 with the TS array. (XLSX)

S7 Table Leading and lagging strand replication gene lists. This table includes the leading and lagging strand gene lists used to define these terms for the analysis in Fig 2 and S1 Fig. *MCM10* was included as a lagging strand replication gene by virtue of its interaction with Pol-α and PCNA [89,93], although a recent study suggested that it has no significant strand bias [79].
(DOCX)

S8 Table List of yeast strains. Yeast strains used in this study with relevant genotypes.
(DOCX)
