# Supplementary figures and images for: Genetic Interactions Implicating Postreplicative Repair in Okazaki Fragment Processing
Source: PLoS Genet. 2015 Nov 6;11(11):e1005659. doi: 10.1371/journal.pgen.1005659 (PMC4636136; doi:10.1371/journal.pgen.1005659)

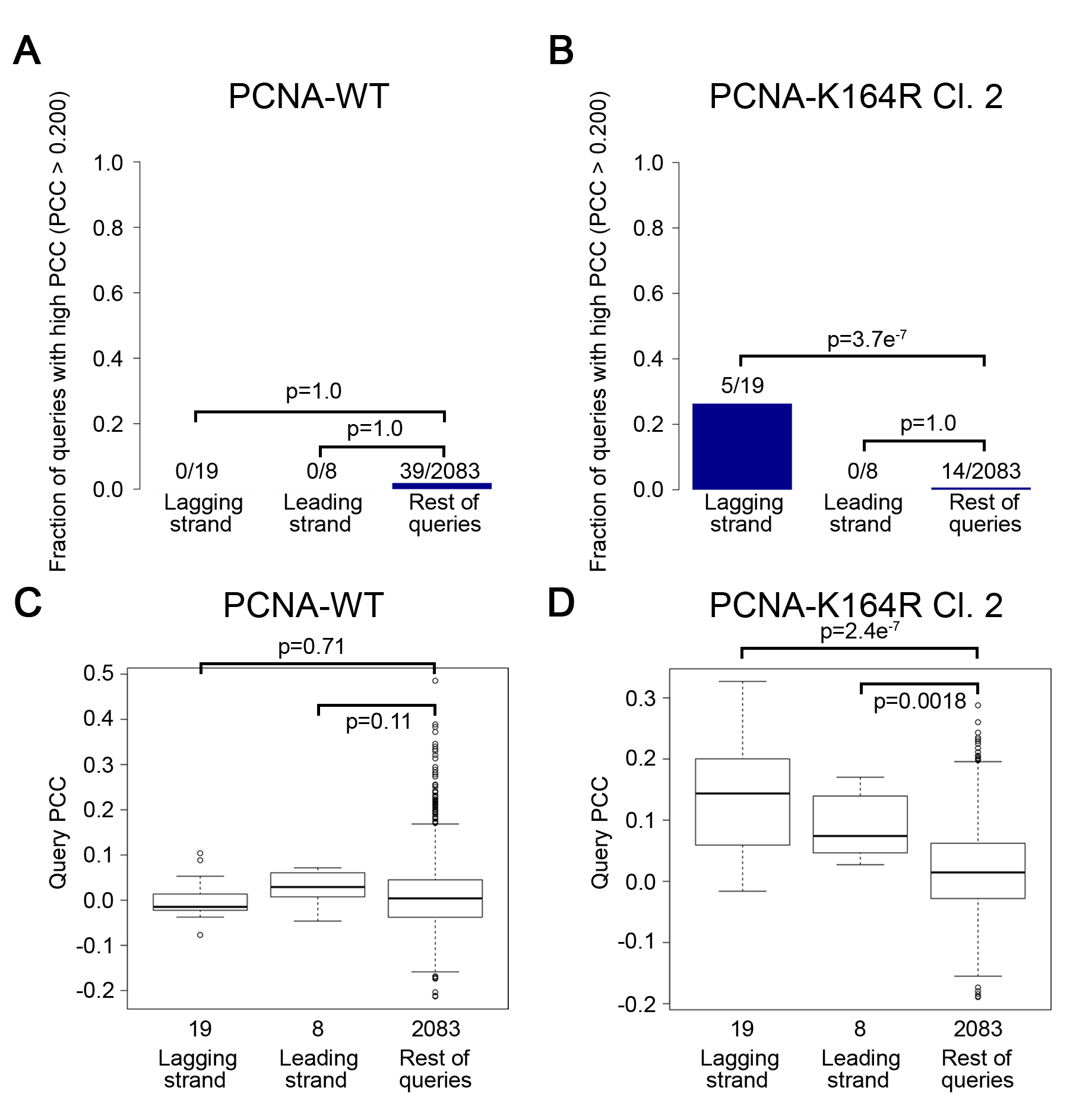

Supplement: S1 Fig — 2 SGA profile, but not that of PCNA-WT, strongly resembles the genetic interactions of lagging strand replication mutants. (A and B) The fraction of leading and lagging strand mutants with a similar profile (PCC > 0.2) to that of the PCNA-WT and PCNA-K164R alleles, respectively. PCNA-K164R Cl.2 was used for this analysis. All mutants were queried against the TS array. Significance was determined using Fisher’s exact test. (C and D) The distribution of profile similarities (calculated using PCC) between PCNA-WT or PCNA-K164R, respectively, and leading or lagging strand replication terms. PCNA-K164R Cl.2 was used for this analysis. All mutants were queried against the TS array. Horizontal lines within the boxes indicate the median PCC. Error bars encompass the middle quartiles of the PCC value distribution. Outliers are represented with circular dots. Significance was determined by the Wilcoxon rank sum test [43]. (TIF) [file pgen.1005659.s001.tif]

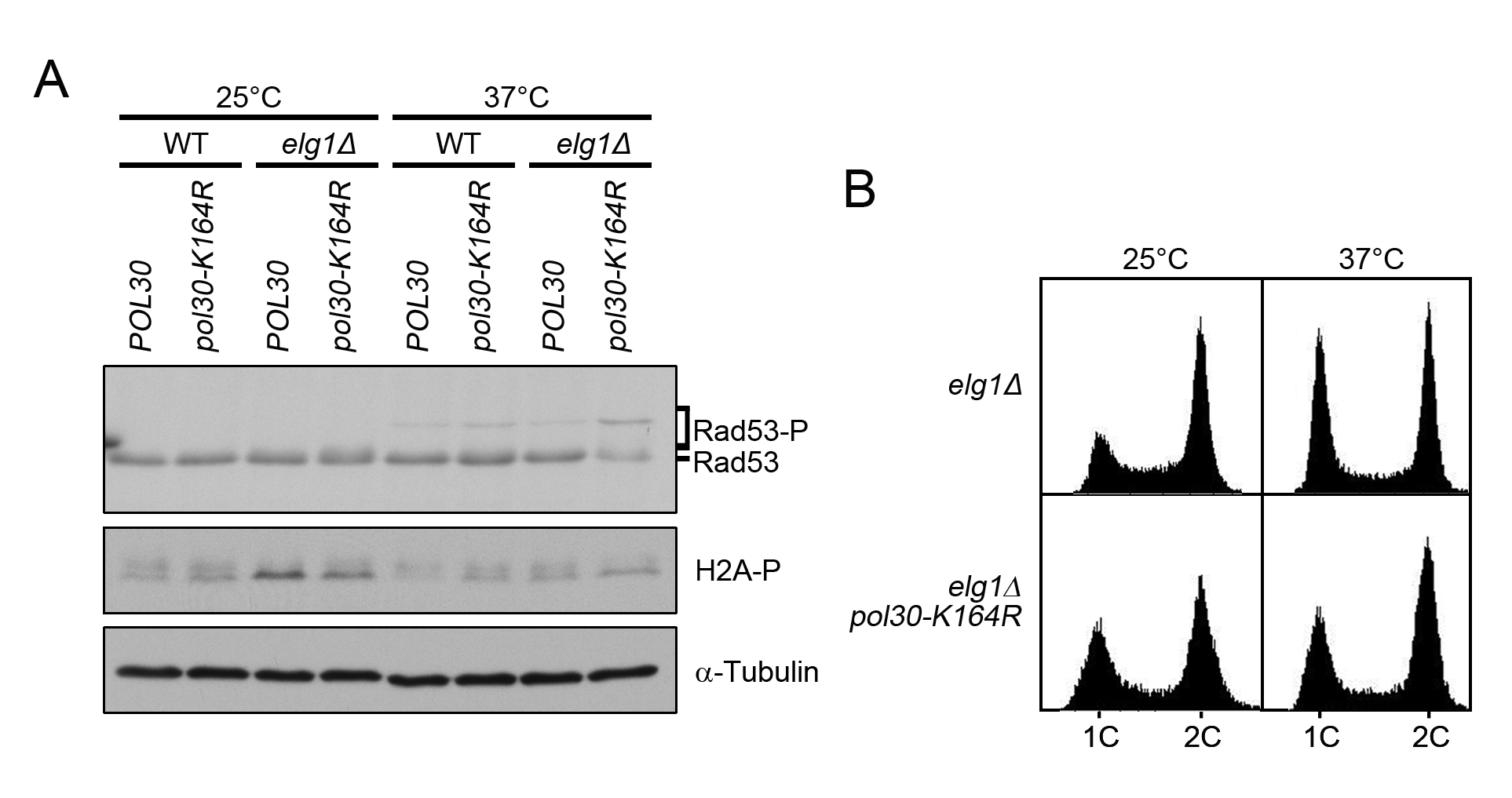

Supplement: S2 Fig — (A) The indicated strains were grown to OD600 = 0.600 at 25°C and then split in half, either remaining at 25°C or being shifted to 37°C. Both cultures were harvested after 3 h growth and protein was extracted by TCA precipitation. Extracts were fractionated by SDS-PAGE and analyzed by western blot with antibodies specific to Rad53 and phospho-H2A-S129. Tubulin served as a loading control. (B) Aliquots of the same cultures from (A) were analyzed for DNA content by flow cytometry. (TIF) [file pgen.1005659.s002.tif]

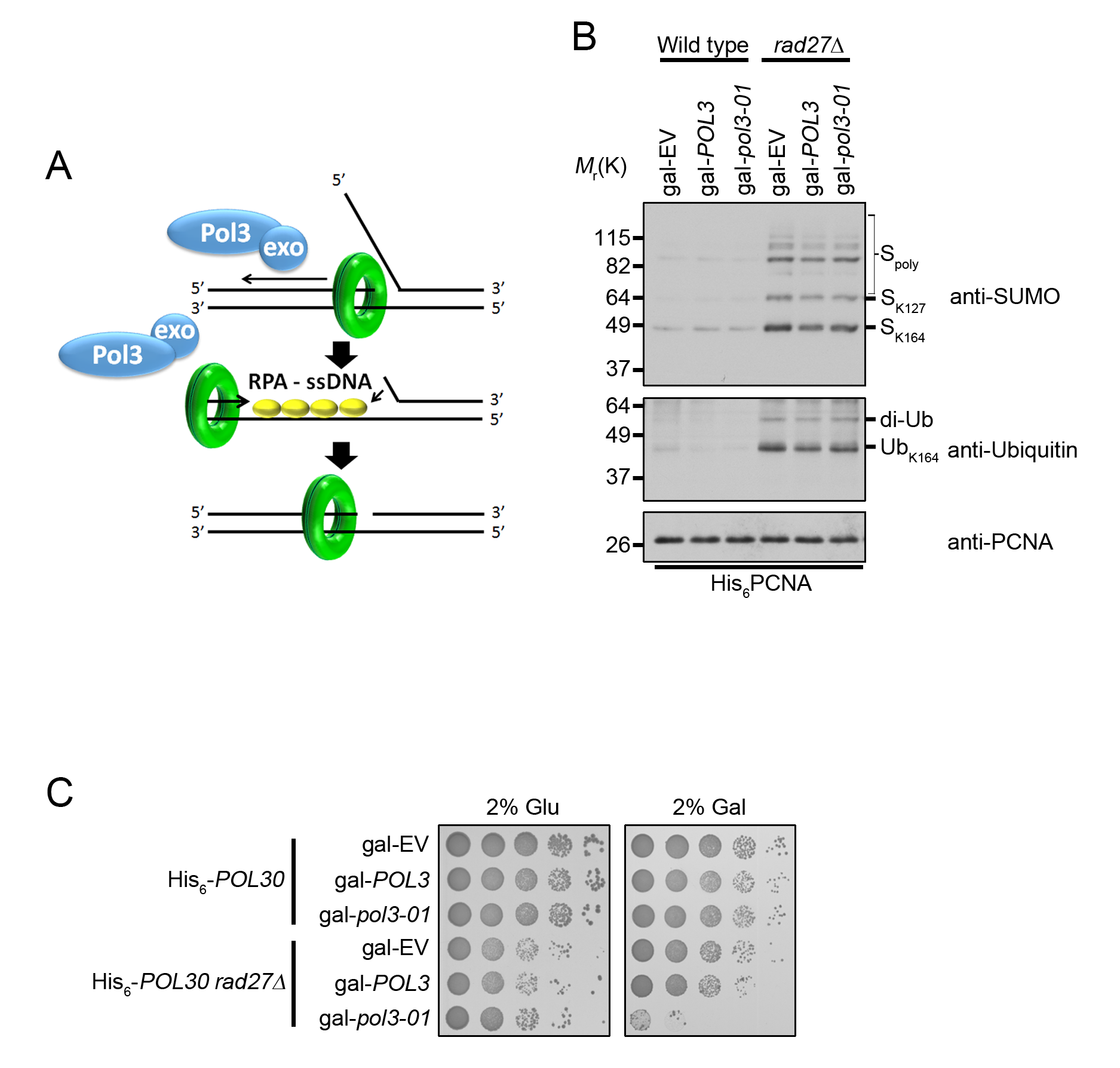

Supplement: S3 Fig — (A) Long flaps generated in the absence of Rad27 are processed into short flaps before Pol3-exo activity resects the 3’ end of the nascent DNA strand allowing for the short flap to re-anneal and form a ligatable nick. If 3’ resection is extensive enough to form a ssDNA region sufficient to bind RPA we considered that this could serve as the stimulus for PCNA ubiquitination in rad27Δ. (B) Wild type and rad27Δ cells carrying gal-EV, gal-POL3, or gal-pol3-01 plasmids were grown to OD600 = 0.600 at 25°C in raffinose containing medium lacking tryptophan. Galactose was then added to a final concentration of 2% and the cultures were shifted to 37°C for 3 h before harvesting. His6-PCNA was purified under denaturing conditions and analyzed by western blot with antibodies specific to PCNA, ubiquitin, and SUMO as indicated. (C) 10-fold serial dilutions of the indicated strains were incubated 3 days at 35°C on medium lacking tryptophan and containing either 2% glucose or 2% galactose. (TIF) [file pgen.1005659.s003.tif]

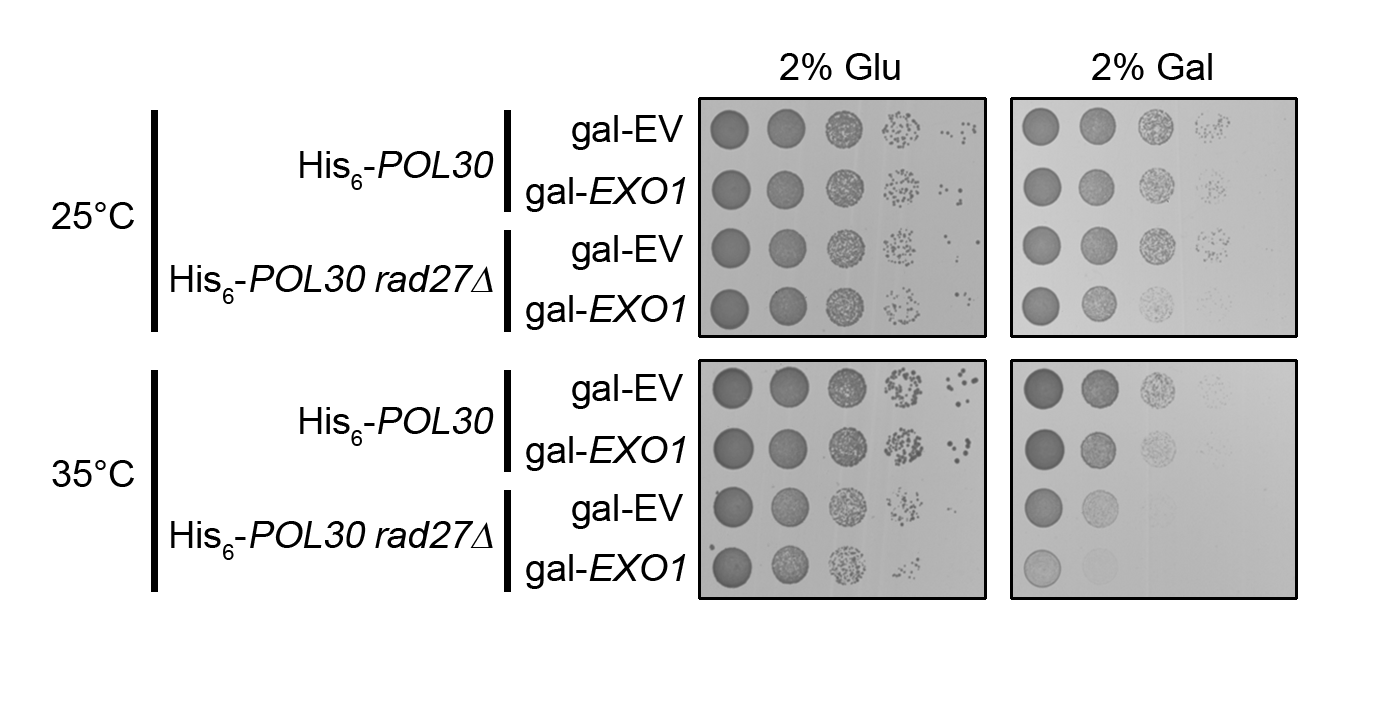

Supplement: S4 Fig — 10-fold serial dilutions of the indicated strains were incubated 3 days at 25°C or 35°C on medium lacking uracil and containing either 2% glucose or 2% galactose. (TIF) [file pgen.1005659.s004.tif]
