# Supplementary material for: Genetic Interactions Implicating Postreplicative Repair in Okazaki Fragment Processing
Source: PLoS Genet. 2015 Nov 6;11(11):e1005659. doi: 10.1371/journal.pgen.1005659 (PMC4636136; doi:10.1371/journal.pgen.1005659)
Supplement: S7 Table — This table includes the leading and lagging strand gene lists used to define these terms for the analysis in Fig 2 and S1 Fig. MCM10 was included as a lagging strand replication gene by virtue of its interaction with Pol-α and PCNA [89,93], although a recent study suggested that it has no significant strand bias [79]. (DOCX) [file pgen.1005659.s011.docx]

| **Leading Strand** | **Lagging Strand** |
| --- | --- |
| *POL2* | *POL1* |
| *DPB3* | *POL12* |
| *MCM2* | *PRI1* |
| *MCM3* | *PRI2* |
| *MCM4* | *MCM10* |
| *MCM5* | *CTF4* |
| *MCM6* | *POL3* |
| *MCM7* | *POL31* |
|  | *POL32* |
|  | *RAD27* |
|  | *DNA2* |
|  | *CDC9* |
|  | *ELG1* |
|  | *POL30* |
|  | *RFC1* |
|  | *RFC2* |
|  | *RFC3* |
|  | *RFC4* |
|  | *RFC5* |

**S7 Table. Leading and lagging strand replication gene lists.**
