# Supplementary material for: Genetic Interactions Implicating Postreplicative Repair in Okazaki Fragment Processing
Source: PLoS Genet. 2015 Nov 6;11(11):e1005659. doi: 10.1371/journal.pgen.1005659 (PMC4636136; doi:10.1371/journal.pgen.1005659)
Supplement: S8 Table — Yeast strains used in this study with relevant genotypes. (DOCX) [file pgen.1005659.s012.docx]

**S8 Table: List of yeast strains used in this study.**

| **Strain Name** | **Relevant Genotype** | **Source** |
| --- | --- | --- |
| EFS20 | *MATα, ade5-1, lys2-A12, trp1-289, his7-2, leu2-3,112, ura3-52, bar1* | Tran *et al*., 1997 |
| AByb1724 | *rad27::kanMX* | This study |
| AByb1733 | *pol30::POL30 (LEU2)* | This study |
| AByb1735 | *pol30::pol30-K164R (LEU2)* | This study |
| AByb1809 | *rad27::kanMX, pol30::POL30 (LEU2)* | This study |
| AByb1810 | *rad27::kanMX, pol30::pol30-K164R (LEU2)* | This study |
| AByb2049 | *rad5::TRP1* | This study |
| AByb2051 | *rad27::kanMX, rad5::TRP1* | This study |
| AByb2053 | *rad18::TRP1* | This study |
| AByb2055 | *rad27::kanMX, rad18::TRP1* | This study |
| AByb2062 | *leu2::His-POL30 (LEU2), pol30::TRP1* | This study |
| AByb2083 | *rev3::LEU2* | This study |
| AByb2085 | *rad27::kanMX, rev3::LEU2* | This study |
| AByb2086 | *rev3::LEU2, rad5::TRP1* | This study |
| AByb2087 | *rad27::kanMX, rev3::LEU2, rad5::TRP1* | This study |
| AByb2169 | *leu2::His-POL30 (LEU2), pol30::TRP1, rad27::URA3* | This study |
| AByb2171 | *leu2::His-pol30-K164R (LEU2), pol30::TRP1, rad27::URA3* | This study |
| AByb2192 | *elg1::TRP1, pol30::pol30-K164R (LEU2)* | This study |
| AByb2193 | *siz1::TRP1* | This study |
| AByb2200 | *elg1::TRP1* | This study |
| AByb2213 | *leu2::His-pol30-K164R (LEU2), pol30::TRP1, elg1::URA3* | This study |
| AByb2233 | *leu2::His-POL30 (LEU2), pol30::TRP1, elg1::URA3* | This study |
| AByb2242 | *rad27::kanMX, leu2::His-POL30 (LEU2), pol30::URA3, pRS424gal-EV (TRP1)* | This study |
| AByb2243 | *rad27::kanMX, leu2::His-POL30 (LEU2), pol30::URA3, pBL336 (TRP1)* | This study |
| AByb2244 | *rad27::kanMX, leu2::His-POL30 (LEU2), pol30::URA3, pBL336-01 (TRP1)* | This study |
| AByb2252 | *leu2::His-POL30 (LEU2), pol30::URA3, pRS424gal-EV (TRP1)* | This study |
| AByb2253 | *leu2::His-POL30 (LEU2), pol30::URA3, pBL336 (TRP1)* | This study |
| AByb2254 | *leu2::His-POL30 (LEU2), pol30::URA3, pBL336-01 (TRP1)* | This study |
| AByb2277 | *leu2::His-POL30 (LEU2), pol30::TRP1, pJS227 (URA3)* | This study |
| AByb2278 | *leu2::His-POL30 (LEU2), pol30::TRP1, gal-DNA2 (URA3)* | This study |
| AByb2281 | *rad27::kanMX, leu2::His-POL30 (LEU2), pol30::TRP1, pJS227 (URA3)* | This study |
| AByb2282 | *rad27::kanMX, leu2::His-POL30 (LEU2), pol30::TRP1, gal-DNA2 (URA3)* | This study |
| AByb2299 | *leu2::His-POL30 (LEU2), pol30::TRP1, pRS316 (URA3)* | This study |
| AByb2300 | *leu2::His-POL30 (LEU2), pol30::TRP1, gal-EXO1 (URA3)* | This study |
| AByb2301 | *leu2::His-pol30-K164R (LEU2), pol30::TRP1, pRS316 (URA3)* | This study |
| AByb2303 | *rad27::kanMX, leu2::His-POL30 (LEU2), pol30::TRP1, pRS316 (URA3)* | This study |
| AByb2304 | *rad27::kanMX, leu2::His-POL30 (LEU2), pol30::TRP1, gal-EXO1 (URA3)* | This study |
| AByb2410 | *rad27::kanMX, siz1::TRP1* | This study |
| AByb2412 | *leu2::His-POL30 (LEU2), pol30::TRP1, gal-exo1-D173A (URA3)* | This study |
| AByb2414 | *rad27::kanMX, leu2::His-POL30 (LEU2), pol30::TRP1, gal-exo1-D173A (URA3)* | This study |
| SSL204 | *MATα, ade2, his3Δ200, trp1, leu2, ura3-52* | Becker *et al*. 2014 |
| ABy2430 | *leu2::His-POL30 (LEU2), pol30::TRP1, pRS316 (URA3)* | This Study |
| ABy2432 | *leu2::His-POL30 (LEU2), pol30::TRP1, gal-EXO1 (URA3)* | This Study |
| ABy2434 | *pol1-1, leu2::His-POL30 (LEU2), pol30::TRP1, pRS316 (URA3)* | This Study |
| ABy2436 | *pol1-1, leu2::His-POL30 (LEU2), pol30::TRP1, gal-EXO1 (URA3)* | This Study |
| ABy2438 | *pol1-1, leu2::His-pol30-K164R (LEU2), pol30::TRP1, pRS316 (URA3)* | This Study |
| ABy2440 | *pol1-1, leu2::His-pol30-K164R (LEU2), pol30::TRP1, gal-EXO1 (URA3)* | This Study |
